# Transcranial Direct Current Stimulation, Symptomatology, and Cognition in Psychosis: A Qualitative Review

**DOI:** 10.3389/fnbeh.2018.00094

**Published:** 2018-05-28

**Authors:** Tina Gupta, Nicholas J. Kelley, Andrea Pelletier-Baldelli, Vijay A. Mittal

**Affiliations:** ^1^Department of Psychology, Northwestern University, Evanston, IL, United States; ^2^Department of Psychology and Neuroscience, University of Colorado Boulder, Boulder, CO, United States; ^3^Department of Psychiatry, Northwestern University, Chicago, IL, United States; ^4^Institute for Policy Research, Northwestern University, Evanston, IL, United States; ^5^Medical Social Sciences, Northwestern University, Chicago, IL, United States; ^6^Institute for Innovations in Developmental Sciences, Northwestern University, Chicago, IL, United States

**Keywords:** transcranial direct current stimulation, tDCS, cognition, neurocognition, symptoms, schizophrenia

## Abstract

Schizophrenia is a chronic, debilitating condition that affects approximately 1% of the population. Individuals diagnosed with schizophrenia typically exhibit positive (e.g., hallucinations) and negative symptoms (e.g., anhedonia) and impairments in cognitive function. Given the limitations of antipsychotic medication and psychotherapy in fully treating psychosis symptomatology, there has been increasing interest in other interventions such as transcranial direct current stimulation (tDCS). tDCS is a non-invasive neuromodulation technique, that is safe, cost-effective, and widely accessible. Here, we discuss treatment studies that seek to improve symptoms and cognitive performance in schizophrenia using tDCS. Currently within the literature, there is support for reductions in positive symptoms such as hallucinations after receiving tDCS. Further, studies indicate that tDCS can improve cognitive functioning, which is an area of investigation that is sorely needed, as it is unclear which types of interventions may be useful in ameliorating cognitive deficits among this group. Taken together, the evidence suggests that tDCS holds promise in improving symptoms and cognition. To that end, tDCS has critical clinical implications for this population.

## Introduction

Schizophrenia is a heterogeneous, chronic condition that affects approximately 1% of the population (Saha et al., 2005). The disorder typically emerges in early adulthood and can have tremendous impacts on an individual’s overall quality of life (Hutchinson et al., 1999; Mueser et al., 2001; Marwaha and Johnson, 2004). Schizophrenia is characterized by positive (e.g., hallucinations and delusions) and negative symptoms (e.g., anhedonia and avolition). In addition, there tends to impairments in cognitive function, particularly in working memory and attention (Schaefer et al., 2013; Fatouros-Bergman et al., 2014; Green and Harvey, 2014).

Efforts to ameliorate symptomatology and cognitive impairments are ongoing, with antipsychotics representing the most commonly used treatment (Tandon et al., 2010). Although antipsychotic medications are effective in reducing symptomatology, they are expensive (Geddes et al., 2000), and can have side effects such as weight gain and fatigue (Young et al., 2015). As such, adherence is often an ongoing challenge and treatment with these medications is generally not considered effective for cognitive function (Young et al., 1986; Fenton et al., 1997; Lacro et al., 2002; Green and Harvey, 2014). Thus, there is a critical need for efficacious treatments that have favorable side-effect profiles, are cost-effective, and address additional impacted domains. Within the last decade, there has been interest in using transcranial direct current stimulation (tDCS), a neuromodulatory technique that releases a weak electrical current through the skull to reduce clinical symptoms and cognitive deficits (Bose et al., 2014; Hoy et al., 2014; Mondino et al., 2015, 2016; Rassovsky et al., 2015; Hasan et al., 2016; Palm et al., 2016). Overall, tDCS is promising, but results remain mixed and future work is needed to understand the efficacy of this technique.

To date, there are informative reviews investigating the impacts of tDCS on schizophrenia populations (e.g., Brunoni et al., 2014; Fregni et al., 2015; Fröhlich et al., 2015; Kadosh, 2015; Mondino et al., 2015) and more recent reviews discussing tDCS and symptoms (Osoegawa et al., 2018; Pontillo et al., 2018). However, there have been several new tDCS studies conducted which are summarized in the current review. The current review discusses (1) recent findings exploring the impacts of tDCS on symptoms and cognition among schizophrenia populations (Fröhlich et al., 2016; Palm et al., 2016), (2) tDCS and other neuromodulatory techniques (Hasan et al., 2016; Hopfinger et al., 2017; Mellin et al., 2018), (3) tDCS and neuroimaging (Mondino et al., 2016; Palm et al., 2016), (4) tDCS in conjunction with cognitive remediation (Orlov et al., 2017), and (5) tDCS and the impacts on antipsychotic medication use (Agarwal et al., 2016). Furthermore, other populations are also discussed including relevant studies in healthy individuals (Khalighinejad and Haggard, 2015; Khalighinejad et al., 2016; Mai et al., 2016; Schülke and Straube, 2017; Straube et al., 2017), childhood onset schizophrenia (Mattai et al., 2011), and non-clinical psychosis (Gupta et al., 2017). The following qualitative review summarizes the current literature and offers future directions, taking recent findings into account, for tDCS research in schizophrenia populations.

## Transcranial Direct Current Stimulation

Transcranial direct current stimulation is a non-invasive brain stimulation technique which modulates cortical excitability by means of a weak electrical current [typically less than 2 milliamps (mA)] traveling between two electrodes. The positively charged anode increases cortical excitability whereas the negatively charged change decreases cortical excitability. Researchers interested in increasing cortical excitability in a target brain region place the anode over that region with the cathode placed over another region irrelevant to the target behavior. This is known as a bilateral montage. In other cases, researchers may place the cathode on an extracephalic region (e.g., shoulder) and this is known as a unipolar montage. Likewise, a bilateral montage aimed at decreasing cortical excitability in a target brain region would place the cathode over the target region with the anode now placed over another region irrelevant to the target behavior. Finally, the positioning of a unipolar montage aimed at decreasing cortical excitability would be the same except for the positioning of the anode and cathode reversed. In addition to active tDCS conditions aimed at increasing or decreasing cortical excitability, most tDCS studies also include a sham condition. The sham condition is a placebo condition where participants receive brief stimulation (approximately 30 s) while all other parameters are held constant. This is a reliable placebo control that does not result in any aftereffects (Gandiga et al., 2006). Sham conditions are increasingly used in study designs (Mattai et al., 2011; Brunelin et al., 2012; Gomes et al., 2015; Smith et al., 2015; Mondino et al., 2016; Palm et al., 2016), and one potential benefit of this condition is the ability to conduct double-blind studies. The ability to conduct sham-controlled double-blind studies is one key strength of tDCS over other brain stimulation techniques.

To date, the precise mechanism of action underlying tDCS remains uncertain. As noted earlier, tDCS involves the use of a weak electrical current (0.5–2 mA) traveling between the anode and cathode (Nitsche et al., 2008; Brunoni et al., 2012). This weak current does not directly cause action potentials as is the case with transcranial magnetic stimulation (TMS). Instead, the current seems to modulate the probability of cell firing without directly triggering an action potential with anodal stimulation increasing and cathodal stimulation decreasing that probability (Nitsche et al., 2008; Agarwal et al., 2013; Mondino et al., 2015). Several animal studies indicate that anodal tDCS causes depolarization by increasing resting membrane potentials, thus leading to spontaneous cell firing (Nitsche and Paulus, 2000; Kekic et al., 2016). In contrast, cathodal tDCS may be decreasing the resting membrane potential leading to a hyperpolarization of neurons.

It is unclear how long the effects resulting from tDCS remain present. Studies indicate that more than 10 min of tDCS can lead to effects lasting over an hour, while shorter durations of tDCS may not have longer after-effects; however, it can vary depending on the region of interest (Nitsche and Paulus, 2000). Furthermore, some studies implement a single session of tDCS, while others utilize multiple sessions (Agarwal et al., 2013; Kekic et al., 2016). Although both approaches are informative, the lasting effects, optimal number of length per session, and duration remain an empirical question (Agarwal et al., 2013; Kekic et al., 2016). While there are aspects of tDCS that have yet to be determined, it is evident that the affordable and portable nature of this neuromodulation technique can have the potential to reach individuals that are more difficult to access (e.g., individuals in more rural locations). Perhaps because of the affordability and portability of tDCS there has been sustained interest in implementing tDCS protocols in clinical populations (e.g., depression, see Brunoni et al., 2016).

## Studies Evaluating the Efficacy of tDCS on Influencing Clinical Symptoms in Psychosis

Hallmark symptoms of schizophrenia include positive symptoms such as hallucinations (e.g., false perceptions such as auditory hallucinations like hearing voices or visual hallucinations such as seeing something that is not there) and delusions (e.g., irrational beliefs such as believing in conspiracy theories or someone is watching, without any reason to believe this). While the development of auditory hallucinations remains unclear, there is evidence indicating abnormal connectivity between the dorsal lateral prefrontal cortex (DLPFC) and temporal parietal junction (TPJ) are linked with auditory hallucinations (Jardri et al., 2011; Mondino et al., 2016). There is also some work indicating that patients diagnosed with schizophrenia exhibit over-activation in brain regions such as left-temporo-parietal areas and left inferior frontal areas (Jardri et al., 2011).

### tDCS and Auditory Hallucinations

The most common montage used when applying tDCS in order to reduce treatment-resistant auditory hallucinations entails placing electrodes over the fronto-temporal network (Mondino et al., 2015). Brunelin et al. (2012) implemented the first study design that was double-blind, randomized, and also included a sham condition in a sample of 30 patients diagnosed with schizophrenia experiencing auditory verbal hallucinations. In this study, 15 patients were randomized to receive active tDCS and 15 patients were assigned to the sham condition. The cathode in this design was placed over the TPJ and the anode was placed over the left DLPFC in order to test whether tDCS could reduce auditory hallucinations. Specifically, active 2 mA tDCS or sham was administered for 20 min, twice daily, for 5 days. Data from the Auditory Hallucination Rating Scale (AHRS; Hoffman et al., 2003) was gathered immediately following the 5 days of receiving tDCS, 1 month, and 3 months later. Findings from the study indicate that auditory verbal hallucinations were robustly reduced by tDCS in the active tDCS condition compared to the sham and importantly, effects lasted up to 3 months. These data are informative because they shed light on the interplay between the DLPFC, TPJ, and auditory hallucinations and provide evidence for the clinical utility of tDCS in both the short and long term and have important lasting effects.

Contrary to the work from Brunelin et al. (2012), null findings have also been reported. For example, in another study using a sample of 24 patients diagnosed with schizophrenia and schizoaffective disorder, the anode was applied over the DLPFC, and the cathode over the temporal parietal area in comparison with a sham condition (Fitzgerald et al., 2014). Active tDCS was applied for 15 daily sessions over three consecutive weeks and outcome measures consisted of the Positive and Negative Syndrome Scale (PANSS) to assess for auditory hallucinations. In contrast to Brunelin et al. (2012), this study did not exhibit changes in reports of auditory hallucinations after receiving tDCS. Differences in findings in the study conducted by Brunelin et al. (2012) and Fitzgerald et al. (2014) could be better explained by the amount of stimulation (twice daily for 5 days vs. once daily for 3 weeks). However, the study did indicate that tDCS was safely implemented and reported minimal side effects and adverse effects providing further evidence for the tolerability of tDCS with patients.

Given the contradictory findings, other groups have attempted to replicate results indicating that tDCS may reduce auditory hallucinations (Bose et al., 2014; Fröhlich et al., 2016). For example, in a study with 21 patients with schizophrenia experiencing persistent auditory hallucinations (even on antipsychotic medications), the anode was applied over the left DLPFC and the cathode over the left TPJ. This study found that tDCS contributed to decreased auditory hallucinations (Bose et al., 2014). Similarly, an exploratory study was conducted using daily tDCS to treat auditory hallucinations in a sample of 26 patients diagnosed with schizophrenia and schizoaffective disorder (Fröhlich et al., 2016). Following similar methods from Brunelin et al. (2012), the group used a randomized, double-blind, sham-controlled study design and applied active tDCS over the left frontal and temporal-parietal areas for 5 days, once a day (in contrast to Brunelin et al., 2012 in which tDCS was applied twice daily) due to difficulties with patient compliance. Results suggest a significant reduction in auditory hallucinations measured by the AHRS in the sham condition. These findings provide information regarding the importance of the design of the study (continuing to work on determining what is considered gold-standard regarding dose) and working toward teasing apart the mechanisms underlying the sham condition given the placebo response that was observed.

### tDCS and Negative Symptoms

There is also research exploring the impacts of tDCS on negative symptoms (Osoegawa et al., 2018; Pontillo et al., 2018). Previous work has examined negative symptoms in conjunction with positive symptoms such as auditory hallucinations, also targeting the DLPFC (Brunelin et al., 2012; Fröhlich et al., 2016). Negative symptoms are a collection of experiences that have been described as a loss of regular functions such as having a lack of motivation to persist in goal-directed activities or emotional blunting (Fusar-Poli et al., 2015). While antipsychotic medications have been impactful in the reduction of positive symptoms, negative symptoms have been suggested to persist (Fusar-Poli et al., 2013). In a recent review, the authors concluded that there is evidence supporting the use of tDCS particularly over prefrontal areas to decrease negative symptoms (Aleman et al., 2018). Brunelin et al. (2012) (described above) also reported decreases in negative symptom scores based off of the PANSS. Similarly, Gomes et al. (2015) conducted a randomized, double-blind study and applied the anode over the left DLPFC and placed the cathode over the right DLPFC. A total of 15 patients with schizophrenia were randomized to either active condition or the sham condition and received 20 min of tDCS once a day for 10 days and found reductions in negative symptom scores (also assessed by the PANSS). Together, there is evidence indicating that tDCS may improve negative symptoms. Future work will benefit from investigating other relevant brain regions and outcome measures in addition to determining the effects of tDCS on specific types of negative symptoms (e.g., anhedonia, avolition).

### Other Neuromodulation Techniques and Clinical Symptoms

More recently, studies have expanded on the current work exploring the nature of tDCS and symptoms but now have integrated other stimulation techniques such as transcranial alternating current stimulation (tACS) (Paulus, 2011). This technique releases a weak electric current similar to tDCS, however, the current targets brain oscillations specific to frequency that is indicated to enhance naturally occurring brain oscillations. Repeated TMS (rTMS) is another stimulation method that has been used within the field, causing the release of new action potentials via activating axons (Aleman et al., 2007). Together, there is evidence for symptom reduction using these approaches within schizophrenia populations (Aleman et al., 2007; Paulus, 2011; Hasan et al., 2016; Hopfinger et al., 2017; Alexander et al., 2018; Mellin et al., 2018).

According to a meta-analysis conducted by Kennedy et al. (2018) (in which seven tDCS RCTs and 30 rTMS RCTs were identified), compared to sham, tDCS improved symptoms with effect sizes around 0.10–0.63. Most notably, higher cumulative stimulation was related to a reduction in auditory hallucinations, while rTMS was not related to cumulative dose, but rTMS was also found to be effective in the treatment of hallucinations. In another study, tDCS and tACS were compared (Mellin et al., 2018). In the first tACS clinical trial for the treatment of symptoms in a psychiatric population, a total of 22 participants with schizophrenia were randomized to receive (twice daily for 20 min for 5 days) sham, 2 mA peak-to-peak tACS or 2 mA tDCS. tACS had the largest reduction in auditory hallucinations scores (measured by the AHRS) compared to sham and tDCS. While the sample size was small in this study, this work provided important initial evidence for the utility of another stimulation approach (tACS). Future work is needed in order to understand similarities and differences of tACS and tDCS as it is unclear what the overlapping mechanisms are of these approaches, which may contribute to the differing effects and findings.

### tDCS and Neuroimaging

There is a particular interest in integrating functional magnetic resonance imaging (fMRI) methodologies in order to understand the mechanism underlying multiple tDCS sessions, and the relationship with symptomatology (Mondino et al., 2016; Palm et al., 2016). For example, Palm et al. (2016) applied tDCS over the prefrontal cortex, where the anode was placed over the left DLPFC and the cathode was placed over the right supraorbital; a sham condition was also included. A total of 20 patients diagnosed with schizophrenia (endorsing predominately negative symptoms) were randomized to 10 sessions of either active or sham. In addition to applying tDCS, participants received fMRI scans pre-and-post the first tDCS session and pre-and-post the tenth tDCS session. Results indicate decreases in the Scale for Assessment of Negative Symptoms (SANS) and PANSS after active tDCS compared to sham. Furthermore, results indicate changes in subgenual cortex and DLPFC connectivity within frontal-thalamic-temporo-parietal networks – areas that have been identified to be related to negative symptoms.

Similarly, in a study conducted by Mondino et al. (2016), 23 patients with schizophrenia who also endorsed treatment resistant auditory hallucinations were randomly assigned to receive 10 sessions of active (2 mA, 20 min) or sham tDCS (two sessions a day for 5 days). Additionally, resting-state functional connectivity of the left TPJ was also examined. Active tDCS reduced auditory hallucinations and this was related to reductions in resting state connectivity between the left TPJ and the left anterior insula.

### Summary

Overall, tDCS has shown some efficacy for symptoms, specifically refractory auditory hallucinations and negative symptoms. A major strength within the existing work is an effort to replicate previous tDCS findings, which is critical in order to truly comprehend the nature of tDCS. There is also promise in utilizing sham conditions in order to compare effects after being in the active condition. Although outcomes remain ambiguous and the impact of multiple versus single sessions is unclear, tDCS is a promising area of research. There is also promise in exploring the use of other neuromodulatory techniques. The fact that symptoms have been unsuccessfully treated in schizophrenia, and tDCS has shown some potential in targeting these domains, provides support for future work that is needed. In particular, further research is needed to target the potential for impact on other symptomatology (e.g., other aspects positive and negative symptoms). See **Table 1** for a summary of noted studies that include information regarding the population, sample size, design of the study, montages/sites, mA, duration/frequency, variables of interest, and key findings.

**Table 1:** Summary of tDCS and clinical symptoms studies discussed.

Author	Population	*N*	Design	Montage/sites	mA	Duration/frequency	Variables of interest	Key findings
Brunelin et al., 2012	Schizophrenia	30	Double-blind, randomized, sham-controlled	Anode over the left DLPFC; cathode over the left TPJ	2	2× a day, 20 min, 5 days	AHRS, PANSS	Reductions in AH scores lasting for 3 months. Decreases in positive and negative symptoms
Bose et al., 2014	Schizophrenia	21	Open-label	Anode over the left DLPFC; cathode over the left TPJ	2	2× a day, 20 min, 5 days	PSYRATS, SAI	Reduction in AHs and insight scores
Fitzgerald et al., 2014	Schizophrenia, Schizoaffective	24	Double-blind, randomized controlled trial	Anode over the DLPFC and cathode over the temporoparietal area	2	1× per day, 20 min, 15 days	PANSS, SANS	No reductions in AHs or other symptoms
Gomes et al., 2015	Schizophrenia	15	Double-blind, randomized, sham-controlled	Anode over the left DLPFC and cathode over the right DLPFC	2	1× per weekday, 20 min, 10 days	PANSS	Improvement in negative but not positive symptoms
Fröhlich et al., 2016	Schizophrenia, Schizoaffective	26	Double-blind, randomized, sham-controlled	An ode over the left DLPFC and cathode over the left TPJ	2	1× per day, 20 min, 5 days	AHRS, PANSS	Reduction in AHs not specific to treatment group. No changes in positive and negative symptom scores
Palm et al., 2016	Schizophrenia	20	Double-blind, randomized, sham-controlled	Anode over the left DLPFC and cathode over the right supraorbital	2	1× per day, 20 min, 10 days	SANS, PANSS	Decreases in SANS and PANSS

*mA, milliamps; DLPFC, Dorsal Lateral Prefrontal Cortex; TPJ, Temporoparietal Junction; AHRS, Auditory Hallucination Rating Scale; PANSS, Positive and Negative Syndrome Scale; SANS, Scale for Assessment of Negative Symptoms; PSYRATS, Auditory Hallucination Subscale of PSYRATS; AHs, Auditory Hallucinations*.

## Studies Evaluating the Efficacy of tDCS on Influencing Cognition in Psychosis

Cognitive deficits contribute vastly to an individual’s every day functioning and can interfere with social and occupational aspects of daily living (Marwaha and Johnson, 2004; Insel, 2010). As noted, traditional approaches such as antipsychotic medication have been ineffective in improving these functions (Marwaha and Johnson, 2004; Green and Harvey, 2014). Some examples of neurocognitive deficits observed in schizophrenia include working memory, attention, inhibition, and executive function (Green et al., 2012; Barch and Sheffield, 2014; Green and Harvey, 2014).

According to Barch and Sheffield (2014), it has been argued that cognitive impairments may be a result of difficulties in representing goal information in working memory that is important for directing behavior; it is suggested that this deficit may be related to processes within the DLPFC. Overall, it may be that there are impairments in context-processing, that is, the way individuals are able to take in prior information from working memory and engage in current processes (Barch and Sheffield, 2014). Within this context, there have been several recent studies using tDCS to target neurocognitive functioning in this population, particularly applying tDCS over the DLPFC (Vercammen et al., 2011; Hoy et al., 2014; Rassovsky et al., 2015; Smith et al., 2015).

### tDCS and Neurocognition

Hoy et al. (2014), in a sample of 18 patients with schizophrenia, applied 20 min of active (1 and 2 mA) tDCS and sham over the left DLPFC to improve working memory performance (measured by the N-back task requiring participants to continuously maintain, update, and recall number sequences). Working memory performance was evaluated immediately following the active or sham treatment, 20 min after, and 40 min after tDCS. Results suggest that there were no improvements in working memory performance after sham or after receiving 1 mA of active tDCS over time. However, there was a significant improvement over time when receiving 2 mA of tDCS, but not immediately after receiving tDCS in contrast with previous work (Nitsche et al., 2008). These data bring to light the importance of considering the dose of stimulation in tDCS designs and the potential for 2 mA of tDCS to be impactful for working memory improvements.

In another study, a randomized double-blind, sham-controlled design was used in order to test the effects of multiple sessions of tDCS (five sessions on consecutive days) in improving neurocognition in a sample of 37 individuals (19 randomized to active tDCS and 18 randomized to sham tDCS) with schizophrenia and schizoaffective disorder (Smith et al., 2015). The anode was placed over the DLPFC and the cathode was placed over the contralateral supraorbital ridge. Neurocognition was assessed using the MATRICS Consensus Cognitive Battery (MCCB; Nuechterlein et al., 2008), a commonly used battery of tasks used for schizophrenia populations. Findings indicate that after being in the active condition, improvements in the MCCB composite score were observed after the fifth tDCS session in addition to working memory and attention scores. These data provide further evidence for the efficacy of tDCS on cognitive performance and particularly show benefits of multiple, consecutive sessions of tDCS.

### tDCS and Social Cognition

Social cognition is a particularly relevant domain of interest given connections with functioning and overall quality of life (Green et al., 2015). Much of what is known about the effects of tDCS on social cognition stems from studies of healthy individuals (Santiesteban et al., 2012; Khalighinejad and Haggard, 2015; Khalighinejad et al., 2016; Mai et al., 2016; Schülke and Straube, 2017; Straube et al., 2017). Social cognition, distinctly different from neurocognitive processes, has been described as the ability to develop mental representations about oneself, others, and interactions between the two (Adolphs, 2001; Fett et al., 2011). Research in this area using tDCS have found that the left angular gyrus plays a fundamental role in the perceptual experience of agency (being in control of an individual’s own actions and the results of these actions) (Khalighinejad and Haggard, 2015). Furthermore, the DLPFC (which was indicated to have implications for individuals diagnosed with depression) has been found to be relevant (Khalighinejad et al., 2016). Studies among healthy groups have also focused efforts on the TPJ (which was discussed above in the context of auditory hallucinations), relevant for social cognition (Santiesteban et al., 2012; Mai et al., 2016). For example, Mai et al. (2016) found, in a sample of 68 healthy adults who received both anodal and cathodal tDCS over the right TPJ, decreases in theory of mind and cognitive empathy after cathodal stimulation. Similarly, in another study, social cognition was improved after receiving anodal stimulation in imitation and perspective-taking tasks (Santiesteban et al., 2012).

Studies from healthy populations, particularly relating to social cognition, can inform research in tDCS and clinical populations through methodology. This is particularly relevant as studies within schizophrenia populations have shown mixed findings for tDCS effficacy. For example, social cognition was assessed in a sample of 36 individuals with schizophrenia (Rassovsky et al., 2015). Specifically, anode (*N* = 12), cathodal (*N* = 12), or sham (*N* = 12) tDCS (for 20 min with tDCS electrodes placed bilaterally over the DLPFC), was administered and then social cognition performance was re-evaluated. Active tDCS improved performance on an emotion identification task; however, changes were not observed in the other three tasks related to managing emotions, social perception, and theory of mind. Furthermore, tolerability of tDCS was assessed and tDCS seemed to be well-tolerated, with only minimal side effects (e.g., itchiness) reported.

### tDCS and Cognitive Remediation

Research has examined the efficacy of tDCS for targeting cognitive deficits in conjunction with other interventions, such as cognitive remediation. For example, in a study conducted by Orlov et al. (2017), a total of 49 participants were asked to complete a baseline assessment of working memory and implicit learning tasks and following, four cognitive remediation training days (days 1, 2, 14, and 56). In day 1 and day 14, participants also received either active (*N* = 24) or sham (*N* = 25) tDCS for 30 min with the anode over the left DLPFC and the cathode over the right supraorbital area. In the active condition, participants showed improvements in working memory performance compared to individuals in the sham condition on day 2 and during follow up on day 56. This study showed the long-term impact of tDCS on working memory improvements.

### Summary

Together, the literature examining tDCS and cognition is quickly growing. Given that cognition can impact functional outcome, it is an important area to target for intervention. The strengths of tDCS and cognition may lie in the design of the studies (e.g., double-blind, randomized, sham-controlled). Work understanding tDCS and social cognition is still mixed but tDCS has shown some efficacy. tDCS has shown promise in conjunction with other treatment approaches such as cognitive remediation, and further evaluation of tDCS as an adjunctive intervention may prove informative. Limitations include the small number of investigations, and to some extent, the exclusive focus on the DLPFC. While studies applying tDCS over the DLPFC are useful, there are other regions of interest that may be relevant to the etiology of psychosis, which may expand our knowledge of tDCS, schizophrenia, and cognition. See **Table 2** for a summary of noted studies that include information regarding the population, sample size, design of the study, montages/sites, mA, duration/frequency, variables of interest, and key findings.

**Table 2:** Summary of tDCS and cognition studies discussed.

Author	Population	*N*	Design	Montage/sites	mA	Duration/frequency	Variables of interest	Key findings
Hoy et al., 2014	Schizophrenia	18	Double-blind, randomized, sham-controlled	Anode over the left DLPFC; cathode over the right supraorbital region	1 2	1× a day 20 min	N-back task	Improvements in working memory 40 min later
Rassovsky et al., 2015	Schizophrenia	36	Randomized, sham-controlled	Anode and cathode bilaterally over the DLPFC	2	1× a day 20 min	MSCEIT FEIT PONS TASIT MCCB	Improvements in emotion identification
Smith et al., 2015	Schizophrenia Schizoaffective	37	Double-blind, randomized, sham-controlled	Anode over the left DLPFC and cathode over the contralateral supraorbital ridge	2	1× a day 20 min 5 days	MCBB	Improvements in MCCB composite score, working memory, and attention
Orlov et al., 2017	Schizophrenia Schizoaffective	49	Double-blind, randomized, sham-controlled	Anode over the left DLPFC and cathode over the right supraorbital area	2	1x on day 1 and day 14 of cognitive training	N-back task; implicit learning task	Improvements in working memory, but not implicit learning

*mA, milliamps; DLPFC, Dorsal Lateral Prefrontal Cortex; TPJ, Temporoparietal Junction; MSCEIT, The Mayer-Salovey-Caruso Emotional Intelligence Test; FEIT, Facial Emotion Identification Test; PONS, Profile of Nonverbal Sensitivity; TASIT, The Awareness of Social Inference Test; MCCB, MATRICS Consensus Cognitive Battery*.

## Future Directions

To date, studies have shown the potential for tDCS in reducing clinical symptoms and improving cognition, despite null findings that have also been reported. Overall, more work is needed to understand many aspects of tDCS. Presently in the literature, the sample sizes are small, and conducting studies with a larger number of participants is critical. Further, tDCS has been tested in other psychiatric populations such as childhood onset schizophrenia (Mattai et al., 2011), and research in this area could benefit from continuing to extend this work so we can fully understand the strengths and limitations of this neuromodulatory technique.

Research could benefit from additional probing around what the optimal number of sessions of tDCS are and period of time (e.g., days, weeks, months). More answers are needed to tease apart appropriate electrode montages and targets that are influenced such as symptoms and cognition. Further, although work indicates that the effects of tDCS may last up to 1 h after (Reis et al., 2009), more research could contribute to our current implementation. Questions still remain regarding the mechanisms of change which one way to determine this is using sham-controlled designs. Understanding the similarities and differences between tDCS and TMS and other techniques may offer perspective regarding strengths and limitations of this approach.

Other than enhancing study design and improving our understanding regarding the mechanism(s) underlying tDCS, future work could also benefit from examining relationships with medication and direct links with psychosis pathophysiology. There has also been some interesting work conducted to determine the influence of antipsychotic drug use on the impact of tDCS among schizophrenia groups (Agarwal et al., 2016) and determining the pathophysiology of psychosis through tDCS (Hasan et al., 2011) of which more work could continue to expand.

A direction in which tDCS may be advantageous is implementing this technique in at-risk populations such as individuals described as representing non-clinical psychosis (NCP). Psychosis, described as the loss of reality, falls on a continuum with some individuals endorsing psychotic disorders such as schizophrenia and individuals on the other endorse infrequent symptoms such as fleeting auditory hallucinations (e.g., hearing their name being called 1–2 times a year or seeing a shadow 1–2 times a year) (van Os, 2002). The distinguishing factors of this group with other psychosis populations are (1) symptoms are infrequent, (2) symptoms are often accompanied by lower distress and impairment and, (3) functioning is more intact (Kelleher and Cannon, 2011). Although there are pronounced differences, NCP individuals tend to share similar vulnerability deficits observed in psychosis populations (e.g., schizophrenia) and clinical high-risk (CHR; individuals considered at imminent risk for developing psychosis) samples such as emotion recognition deficits (Pelletier et al., 2013), and procedural learning impairments (van Os, 2002; Kelleher and Cannon, 2011; Mittal et al., 2012). Our group investigated whether tDCS could improve procedural learning deficits given studies indicating impairments in these processes among NCP (Mittal et al., 2012; Lunsford-Avery et al., 2017) and CHR samples (Dean et al., 2014). Specifically, using a double-blind, randomized, sham-controlled design, we investigated, in a sample of 18 controls and 24 NCP individuals, whether cerebellar tDCS could improve procedural learning (using a pursuit rotor task) in the NCP group and normalize performance to the level of controls (Gupta et al., 2017). Participants were randomized to receive 25 min of active cerebellar tDCS or sham on separate laboratory visits, 1 week apart. After being in the sham condition, NCP individuals had significantly lower procedural learning performance compared to the control group. However, after being in the active condition, the NCP group normalized procedural learning performance to the level of controls. These data highlight the possibility of using NCP as an analog sample within this work. Furthermore, these findings continue to disentangle the complexities regarding the pathogenesis of psychosis in efforts to develop targeted treatment interventions such as tDCS in order to prevent further symptom decline.

Additional research applying tDCS among individuals at CHR for developing psychotic disorders such as schizophrenia would be also beneficial. It is suggested that about 10–35% of individuals within this group may go on to develop the disorder (Fusar-Poli et al., 2012) and tDCS may be a way to intervene. Studies within this group can contribute to our knowledge regarding psychosis more broadly and also inform the development of targeted treatment interventions as such. Utilizing risk samples may be a first step in extending the present work among schizophrenia populations in order to determine if this technique can provide clinical utility as an intervention approach.

## Author Contributions

All authors contributed to the drafting and editing of the manuscript.

## Conflict of Interest Statement

VM is a consultant to Takeda Pharmaceuticals. The remaining authors declare that the research was conducted in the absence of any commercial or financial relationships that could be construed as a potential conflict of interest.
